# Uptake in cancer screening programmes: a priority in cancer control

**DOI:** 10.1038/sj.bjc.6605391

**Published:** 2009-12-03

**Authors:** D P Weller, C Campbell

**Affiliations:** 1 Division of Community Health Sciences – General Practice, University of Edinburgh, 20 West Richmond St, Edinburgh EH8 9DX, UK

**Keywords:** cancer screening, uptake, inequalities, earlier diagnosis, access

## Abstract

Achieving adequate levels of uptake in cancer screening requires a variety of approaches that need to be shaped by the characteristics of both the screening programme and the target population. Strategies to improve uptake typically produce only incremental increases. Accordingly, approaches that combine behavioural, organisational and other strategies are most likely to succeed. In conjunction with a focus on uptake, providers of screening services need to promote informed decision making among invitees. Addressing inequalities in uptake must remain a priority for screening programmes. Evidence informing strategies targeting low-uptake groups is scarce, and more research is needed in this area. Cancer screening has the potential to make a major contribution to early diagnosis initiatives in the United Kingdom, and will best be achieved through uptake strategies that emphasise wide coverage, informed choice and equitable distribution of cancer screening services.

Cancer screening is an important element of any national strategy to improve early cancer diagnosis. The NAEDI initiative (Department of Health NAEDI Newsletter, 2009) emphasises a multi-faceted approach; there is a need to raise awareness of cancer symptoms and make health services better equipped to respond to symptoms. In parallel, effective and efficient cancer screening programmes based on sound evidence are needed. Indeed, cancer screening can effectively complement awareness and early diagnosis initiatives; a screening programme can, for example, raise awareness in the community of particular cancers and their associated symptoms.

Uptake (i.e. the proportion of screening invitees in a given year for whom a screening test result is recorded) is the most important factor in determining the success of a screening programme (Barratt *et al*, 2002; Parkin *et al*, 2008). The United Kingdom has, over recent decades, taken an organised, population-wide approach to screening; although the exact contribution of cancer screening to an observed reduction in mortality in the population can be difficult to quantify, modelling exercises suggest that cancer screening activity over recent decades has made a significant difference (Blanks *et al*, 2000; Raffle *et al*, 2003; Taylor *et al*, 2004; van der Aa *et al*, 2008). However, mortality reductions are brought about by many contributory factors and therefore combined approaches that include earlier presentation, more timely diagnosis and improved treatments are considered to hold the greatest promise of improved cancer outcomes (Department of Health NHS Cancer Plan, 2000).

Experience in the United Kingdom suggests that achieving and maintaining uptake in cancer screening requires ongoing effort; coverage (i.e. the proportion of resident and eligible individuals who have had a test with a recorded result in a screening round) can decline from one round of screening to the next, and the causes of this drop-off are often difficult to identify (Weller *et al*, 2007; Lancuck *et al*, 2008). A number of systematic reviews have examined the evidence supporting various approaches to cancer screening uptake (Jepson *et al*, 2000; Bonfill *et al*, 2001; Forbes *et al*, 2002). The findings typically vary according to factors such as cancer site, type of test and target population. However, *organised* recruitment strategies (the approach typically taken in the United Kingdom, which involves systematic recruitment and follow-up, the provision of scheduled appointments for screening), and *personalised* invitation approaches (for example, from a general practitioner) seem to show consistent benefits over opportunistic approaches to screening. Reducing structural barriers to accessing services (e.g. location, timing of appointments, childcare facilities) can also increase uptake (Baron *et al*, 2008).

It is timely, in this *BJC* issue, to examine the main predictors of uptake for the United Kingdom's three established cancer screening programmes (breast, cervical and colorectal screening), along with the evidence supporting strategies to improve uptake in each of these programmes.

## Cervical screening

### Variations in uptake

Recently, declining rates in cervical screening, particularly in younger women, have prompted renewed interest in the issue of adequate, population-wide coverage (Lancuck *et al*, 2008). Women from ethnic minorities and deprived sub-groups in the population have shown consistently lower uptake over decades of screening in countries worldwide (Webb *et al*, 2004; Moser *et al*, 2009) – particularly among groups born outside their host country (Downs *et al*, 2008). Health literacy is also an important determinant; the messages in cervical cancer screening recruitment materials are relatively complex, and strategies targeting invitees with limited health literacy require careful design and, ideally, flexibility in recruitment procedures (Giordano *et al*, 2008).

### Strategies to improve uptake

While setting recruitment targets in UK primary care can lead to improved uptake, even in deprived populations (Baker and Middleton, 2003), interventions of proven sustainable benefit are typically multi-faceted, and result from efforts to make the approaches to target groups socially and culturally appropriate (Dietrich *et al*, 2006; Tu *et al*, 2006). The media has an important role to play in cervical screening through, for example, programme story-lines where a character has cancer (Meissner *et al*, 2004) in the United Kingdom this can produce profound short-term effects, although evidence for effectiveness in bringing about sustained increases is variable (Howe *et al*, 2002).

On-going effort, particularly targeting ethnic sub-groups, seems justified in the United Kingdom, although an ‘off-the-shelf’ strategy remains elusive. Ideally, detailed socio-demographic and ethnic information should be available at local levels, to tailor strategies with local effect. The advent of HPV vaccination introduces a new dynamic into cervical screening uptake, and this will require further refinement of messages promoting uptake of screening over the next several years.

## Breast screening

### Variations in uptake

Socio-economic status is a powerful driver of uptake in breast screening, as shown in the recent National Statistics Omnibus Survey (Moser *et al*, 2009). Importantly, deprived populations seem to have later stage presentation in breast cancer-compounding the potential effects of inequalities of breast screening uptake (Cuthbertson *et al*, 2009). There is compelling evidence that certain ethnic sub-groups in the United Kingdom have lower participation rates than the general population (Szczepura *et al*, 2008).

### Strategies to improve uptake

In common with other forms of screening, individuals vary in their response to a breast screening invitation; factors such as perceived risk of cancer and perceived ability to undertake the test are important, and interventions should ideally incorporate theoretical psychological models (Champion *et al*, 2007). Endorsement of breast screening from primary care seems to have a consistent, albeit modest, effect on recruitment (Bankhead *et al*, 2001). Research on targeting low-uptake groups has typically used customised approaches and, on balance, shows a beneficial effect – for example, strategies such as tailored telephone counselling seem to improve uptake (Luckmann *et al*, 2003). We should, however, be cautious about attempting more intense recruitment strategies across different regions of the United Kingdom – again, there is no one strategy with proven benefit in different contexts. While strategies such as phone calls and home visits can produce modest increases in breast cancer screening uptake, these efforts are labour intensive and may not be cost-effective on a population-wide scale in the United Kingdom (Kearins *et al*, 2009).

## Colorectal screening

### Variations in uptake

Colorectal screening, using the FOBT, is now being ‘rolled out’ in all four countries of the United Kingdom. There is already compelling evidence that certain ethnic sub-groups in the United Kingdom have lower participation rates of colorectal screening than the general population – this applies to screening using both FOBT and flexible sigmoidoscopy (Robb *et al*, 2008; Szczepura *et al*, 2008). The reasons for these differences are complex, underpinned by a range of health beliefs and cultural attitudes; in some ethnic sub-groups participation in screening is at odds with cultural norms, and issues such as perceived lack of self-efficacy, cultural misunderstandings, barriers relating to spiritual beliefs and fear of cancer are all significant. Further, profound differences in uptake rates between socio-economic groups have emerged in countries adopting screening (Weller *et al*, 2007; Deutekom *et al*, 2009).

Colorectal screening is targeted at both males and females, and there is some evidence that males and younger age groups have lower uptake rates of FOBT screening (Weller *et al*, 2007; Pornet *et al*, 2009). However, the evidence is less clear for endoscopic procedures such as colonoscopy or flexible sigmoidoscopy (UK Flexible Sigmoidoscopy Screening Trial, 2002; Meissner *et al*, 2006). Men appear to have higher rates of participation, although there are exceptions, such as a recent population-based trial of flexible sigmoidoscopy screening from Norway (Hoff *et al*, 2009).

Again, literacy is a critical determinant of participation in colorectal cancer screening. A great deal of effort has been devoted to producing information and materials for the bowel cancer screening programmes, which are comprehensive, informative and balanced – yet we know that health literacy varies a great deal in the population (von Wagner *et al*, 2009b), and many invitees will have limited comprehension of recruitment materials.

### Strategies to improve uptake

Again, interventions of proven benefit targeting deprived and ethnic sub-groups are typically multi-faceted, and result from efforts to make the approaches to target groups socially and culturally appropriate (Tu *et al*, 2006). The type of test used is also important. On the whole, tests that are simpler and which minimise factors such as cost, inconvenience and distaste (e.g. from handling faeces in faecal occult blood testing), are associated with greater uptake (Federici *et al*, 2005). Endorsement of cancer screening from primary care can improve uptake although, again, the effect is quite modest (Federici *et al*, 2006); evidence from the United States suggests that engagement of wider primary care teams is particularly promising (Hudson *et al*, 2007; Klabunde *et al*, 2007). Low primary care use has been associated with a lack of CRC screening among both women and men (Fenton *et al*, 2009).

As the current programme is implemented screening providers need to avoid the possibility of patterns of low uptake in ethnic and deprived groups becoming entrenched. We have the opportunity with a new programme to trial new, tailored and culturally appropriate approaches to these groups, and there is currently a great deal of research activity and innovation in this area. It is less clear whether specific effort should be directed at males as the differences in uptake are not large and appear to be less consistent in different regions of the United Kingdom. Nevertheless, methods of approach, which accommodate male perspectives and attitudes towards preventive health services, should be considered – these may differ considerably from those of women (Cullati *et al*, 2009), and require specific strategies to overcome gender-specific barriers to screening uptake.

The timing of invitations may also be worth considering – although it would require a substantial re-arrangement of current recruitment processes in the bowel screening programmes. It seems timing can have a positive effect, if invitations coincide with annual milestones such as birthdays and festivals (Hoff and Bretthauer, 2008). We so far have limited evidence supporting the use of web-based resources in promoting CRC screening in the United Kingdom, although US evidence suggests these approaches show promise, particularly, if they have a strong interactive component (Ruffin *et al*, 2007).

## Summary

While other factors are important, ethnicity, social deprivation and gender are the major determinants of cancer screening uptake in United Kingdom cancer screening programmes – their effect on uptake is summarised in Table 1. Strategies to improve cancer screening uptake vary according to context, type of test and target group – a summary of evidence is shown in Table 2. There is a strong programme of research into cancer screening uptake in the United Kingdom, and new evidence is emerging; in this *British Journal of Cancer* supplement von Wagner *et al* (2009a) describe socio-economic patterns of uptake for FOBT screening in London. This adds to a growing body of literature showing that we need to invest extra efforts in reducing inequalities in cancer screening uptake. Further Eilbert *et al* (2009) provide an interesting model for improving uptake – carefully tailored to local context in their region of London, and involving multi-faceted approaches. Increasingly, these are the kinds of approaches we will need to improve uptake in an equitable way.

Ideally, the achievement of adequate uptake rates in cancer screening should be accompanied by *informed* uptake – that is, invitees should make informed choices and be aware of all the risks and benefits of participation (Jepson *et al*, 2005). It is a difficult concept to measure, and involves more than provision of information – it includes a need to check understanding and to explore decision making processes (Jepson *et al*, 2007; Woodrow *et al*, 2008). Increasingly, individuals invited to screening are encouraged to access and use more information to reach decisions over participation (Welch, 2004).

## Conclusions

The reduction in health inequalities is a major feature of the Cancer Reform Strategy. Yet uptake of cancer screening is not uniform across the population – within the United Kingdom, variation in uptake in cancer screening programmes is observed in different population sub-groups: for both breast and cervical screening uptake is lower in deprived communities and in some black and ethnic minority communities; these same patterns are seen in the more recently introduced bowel screening programme, where gender has also been shown to affect participation.

Cancer screening uptake improvement strategies will continue to require careful and creative design; socio-demographic and cultural patterns of cancer screening uptake are well established, and though strategies for improving uptake differ according to the dynamics of the screening process, there are many generic lessons that are transferable between the different screening programmes. There is, for example, a small but growing body of evidence to shape strategies targeting ethnic and deprived populations, and both age- and gender-based sub-groups; however, there is a need to develop a robust evidence base for effective interventions for diverse populations within the United Kingdom.

Cancer screening should ideally work in tandem with other awareness and early diagnosis initiatives. The NAEDI initiative is part of a major push to further improve the United Kingdom's performance in cancer-related health outcomes (Department of Health Cancer Reform Strategy, 2007); cancer screening programmes with good coverage, well-informed invitees and equitable engagement of the population can have a major role.

## Conflict of interest

The authors declare no conflict of interest.

## Figures and Tables

**Table 1:** Effect of deprivation, ethnicity and gender on uptake: examples from UK cancer screening programmes^a^

	**Breast**	**Cervical**	**Colorectal**
Deprivation (range from most deprived to least deprived)	64–81%^b^	60–80%	37.2–61.2%^c^
Ethnicity (range from other/white)	51.7–77.3%^d^	37–75%	21.4–61.3%^d^
Gender	N/A	N/A	48% (M), 56% (F)^c^

a Where figures are derived from published screening programme data, references are shown; otherwise figures are based on unpublished data and reports and represent an estimate of contemporary rates across the United Kingdom.

b Information and Statistics Division Scotland http://www.isdscotland.org/isd/1623.html.

c Weller *et al*, 2007; Steele *et al*, 2009.

d Szczepura *et al*, 2008.

**Table 2:** Summary of interventions with some evidence of effectiveness in cancer screening

	**Breast**	**Cervical**	**Colorectal**
Deprived	Lay worker/patient navigator Telephone counselling Tailored interventions (print and telephone)	Primary care endorsement Workplace initiatives	Largely organisational approaches (in the United States) Simpler tests Telephone support Patient navigators
			
Ethnic	Translation services Community-based interventions (e.g. health educator in a group setting)	Translation services Lay workers Psycho-educational counselling Culturally sensitive materials Home visits	Video and culturally sensitive educational materials Telephone support
			
Gender	N/A	N/A	Little available of proven effectiveness specifically for men

*Notes:*

The table is derived from ‘scoping’ the literature, not a systematic literature review.

It includes information from many non-UK publications, therefore results may not be directly transferable to UK contexts.

There is considerable overlap between population sub-groups, so interventions targeted at one community often straddle for example both ethnicity and deprivation.

Not all studies are equally methodologically robust, and some have small numbers.

The size of effect seen in these studies varies – some improvements were very modest, and there is little evidence regarding how sustained their impact: interventions in the future will likely need to be multi-faceted and address attitudinal, language and cultural concerns.

There is a need for both additional systematic reviews in this area (focused on particular approaches and/or cancer type), and for new research studies in differing UK contexts.
